# Stranding Events of *Kogia* Whales along the Brazilian Coast

**DOI:** 10.1371/journal.pone.0146108

**Published:** 2016-01-05

**Authors:** Jailson F. Moura, Esteban Acevedo-Trejos, Davi C. Tavares, Ana C. O. Meirelles, Cristine P. N. Silva, Larissa R. Oliveira, Roberta A. Santos, Janaína C. Wickert, Rodrigo Machado, Salvatore Siciliano, Agostino Merico

**Affiliations:** 1 Leibniz Center for Tropical Marine Ecology - ZMT, Bremen, Germany; 2 Laboratório de Ciências Ambientais, Universidade Estadual do Norte Fluminense – UENF, Programa de Pós-Graduação em Ecologia e Recursos Naturais, Campos dos Goytacazes, RJ, Brazil; 3 Escola Nacional de Saúde Pública – ENSP, Departamento de Endemias, Fundação Oswaldo Cruz – Fiocruz, Grupo de Estudos de Mamíferos Marinhos da Região dos Lagos - GEMM-Lagos, Rio de Janeiro, RJ, Brazil; 4 Associação de Pesquisa e Preservação de Ecossistemas Aquáticos - AQUASIS, SESC Iparana, Caucaia, CE, Brazil; 5 Laboratório de Ecologia de Mamíferos, Universidade do Vale do Rio dos Sinos - UNISINOS, São Leopoldo, RS, Brazil; 6 Grupo de Estudos de Mamíferos Aquáticos do Rio Grande do Sul – GEMARS, Osório, RS, Brazil; 7 Centro de Pesquisa e Gestão dos Recursos Pesqueiros do Litoral Sudeste e Sul – CEPSUL, Instituto Chico Mendes de Conservação da Biodiversidade - ICMBio, Itajaí, SC, Brazil; 8 Laboratório de Sistemática e Ecologia de Aves e Mamíferos Marinhos – LABSMAR, Universidade Federal do Rio Grande do Sul – UFRGS, Departamento de Zoologia, Porto Alegre, RS, Brazil; 9 Universidade Federal do Rio Grande do Sul – UFRGS, Pós-Graduação em Biologia Animal, Porto Alegre, RS, Brazil; 10 Faculty of Physics & Earth Sciences, Jacobs University, Bremen, Germany; New York Institute of Technology College of Osteopathic Medicine, UNITED STATES

## Abstract

The genus *Kogia*, which comprises only two extant species, *Kogia sima* and *Kogia breviceps*, represents one of the least known groups of cetaceans in the global ocean. In some coastal regions, however, stranding events of these species have been relatively common over the last decades. Stranding provides the opportunity to investigate the biology of these cetaceans and to explore the epidemiological aspects associated with the mortality of the organisms found on the beach. A number of disturbances (including pelagic fisheries, chemical pollution, boat strikes, and noise pollution) have been confirmed to pose a particular threat to the *Kogia* species. However, no study has yet investigated potential relationships between environmental conditions and stranding events. Here we analyse how a collection of environmental, physical, and biological variables, such as wind, sea surface temperature (SST), water depth, and chlorophyll-a, correlate to *Kogia* stranding events along the Brazilian coast. The results of our statistical analyses suggest that *K*. *sima* is more likely found in warm tropical waters, which provide an explanation for the high frequency of stranding in northeastern Brazilian coast. In contrast, *K*. *breviceps* appears to have a preference for temperate and productive waters. Wind speed results to be also an important factor for predicting *Kogia* strandings in Brazilian coast. Additionally, literature information in combination with our own data and analyses of stomach contents confirms that oceanic cephalopods constitute the primary nutritional source of both *Kogia* species. By using the available information as a qualitative proxy for habitat preference and feeding ecology, our study provides a novel and comprehensive assessment of *Kogia* stranding data in relation to environmental conditions along the Brazilian coast.

## Introduction

The genus *Kogia* comprises only two extant species, the pygmy sperm whale (*Kogia breviceps*, de Blainville 1838) and the dwarf sperm whale (*Kogia sima*, Owen 1866). These two species are considered different since 1966 after extensive morphological analyses of cranium, skeleton and external characteristics have pinpointed relevant taxonomic dissimilarities between them [1]. Subsequent studies based also on morphological and molecular analyses have confirmed this taxonomic classification [2–4].

Dwarf and pygmy sperm whales inhabit offshore tropical, subtropical and temperate waters of the world ocean [4]. However, *K*. *sima* appears to prefer warm waters, while *K*. *breviceps* is present in both tropical and temperate waters [2,4,5]. According to Caldwell and Caldwell [5], both *Kogia* species inhabit oceanic waters near continental shelves and slopes, albeit *K*. *breviceps* can be also found further off shore.

Although rarely observed at sea due to their oceanic habits and inconspicuous behaviour [6], estimates in the western North Atlantic and northern Gulf of Mexico suggest alarmingly small populations in these regions, about 850 individuals when considering both species combined [7]. In light of these small numbers, anthropogenic threats such as pelagic fisheries, chemical pollution, boat strikes, and noise pollution [8,9] represent a risk for the long-term viability of these species. Moreover, as apex predators with low reproductive rates, marine mammals are very sensitive to human-driven perturbations [10].

Given the difficulties to observe these whales in their natural habitats, the examination of stranding events represent a valuable opportunity not only to investigate the causes of mortality but also to better understand their ecology [8]. In fact, most of the information on the ecology and biology of these species has been inferred from stranding events [4,11,12].

The monitoring of stranding events is conducted in many coastal environments around the world, including Brazil, and represents an effective approach for investigating the ecology and epidemiology of marine mammals [8,13]. If analysed carefully, for example by minimising inherent biases, stranding data may provide relevant information on the status, distribution, and seasonal abundance of marine mammals and the potential links with environmental changes [14–17].

Most of the existing knowledge about the distribution of *Kogia* species is based on descriptive assessments of whales washed ashore. However, no study has yet investigated the potential relationships between environmental variables and the spatial distribution of strandings events. Understanding how environmental variables can potentially contribute to strandings of marine mammals is relevant for conservation and management efforts, especially in the current context of rapid environmental degradation and climate change [14,16,18,19].

Along the Brazilian coast, the *Kogia* species are among the least known cetaceans. Muñoz-Hincapié et al. [20] reviewed stranding data from South America, and found only 18 occurrences in Brazil between 1965 and 1997. Since 1998, more stranding events have been recorded through beach monitoring programs [21–29]. Studies on feeding ecology and based on the assessment of stomach contents worldwide, indicate that the *Kogia* feed primarily on deep-water organisms, mainly cephalopods, and to a less extent on fish and shrimps [5,30]. Santos and Haimovici [23] analysed the stomach content of *Kogia* specimens stranded in Brazil and obtained results corroborating the preference for oceanic cephalopods.

Here we investigate how a number of environmental, physical, and biological variables, such as wind, sea surface temperature, ocean topography, and chlorophyll-a, contribute to *Kogia* stranding events along the Brazilian coast and analyse information on habitat preferences and dissimilarities between these species. Moreover, we explore the spatial and temporal distribution of stranding and provide relevant information about feeding habits.

## Materials and Methods

### Study area

**Fig 1:**
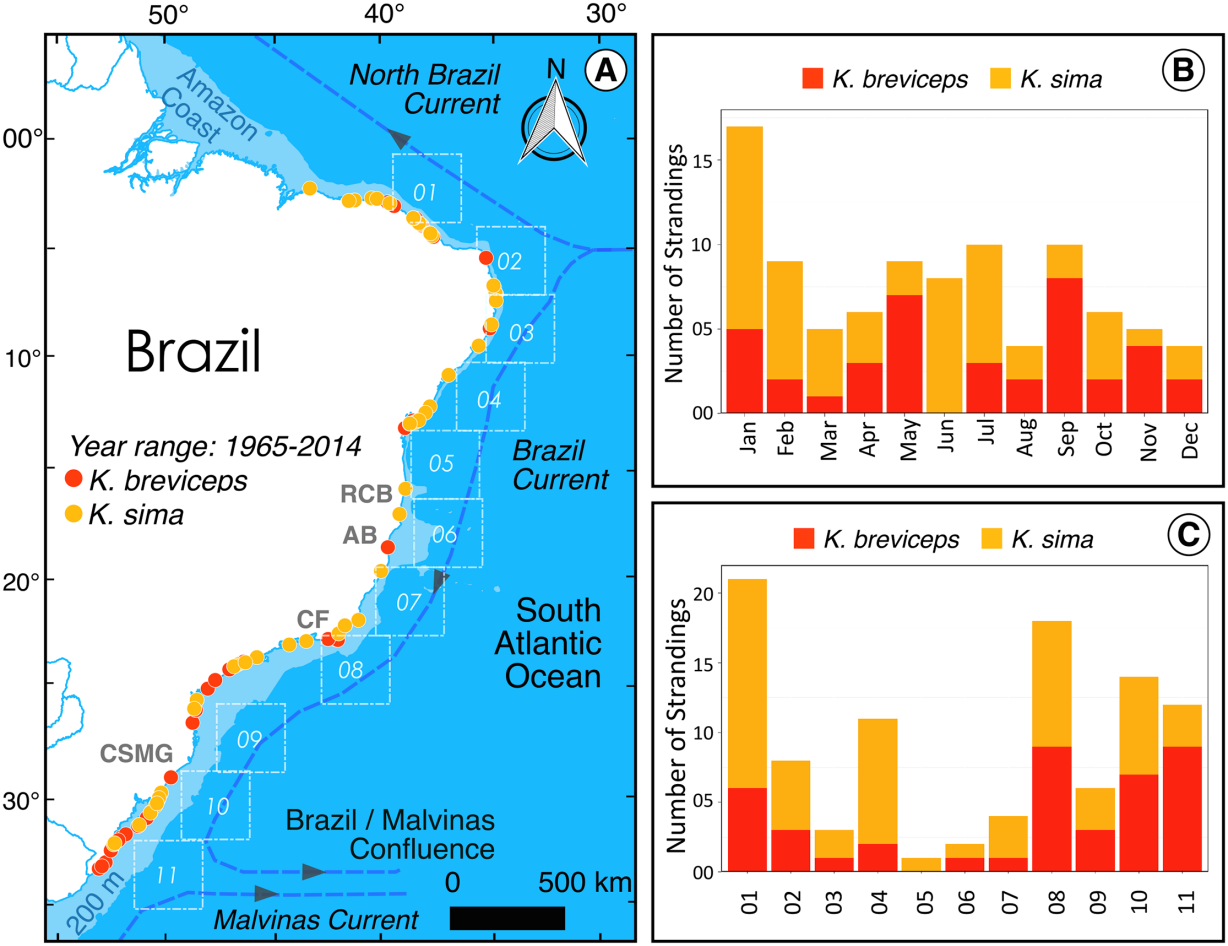
Synoptic map. Panel A shows the spatial distribution of stranding events of the *Kogia* whales (42 and 58 for *K*. *breviceps* and *K*. *sima*, respectively) from 1965 to 2014, the latitudinal sampling boxes (dashed white squares), from where we extracted mean values of environmental variables, adjacent to stranding zones, and important sites (RCB = Royal Charlotte Bank; AB = Abrolhos Bank; CF = Cabo Frio; CSMG = Cabo de Santa Marta Grande). Panel B shows the monthly occurrences of stranding events and panel C shows the number of strandings adjacent to the sampling boxes.

The study area comprises the entire Brazilian coast, about 9.200 km of coastline situated between latitudes 5°N and 34°S (Fig 1). The Brazilian coast is influenced by three main current systems: the Northern Brazil Current (NBC), the Brazil Current (BC) and the Malvinas Current (MC) [31]. The NBC and BC originate by the bifurcation of the westward trans-Atlantic South Equatorial Current, at about 10°S. The NBC transports warm subtropical waters northward along the Brazilian coast, across the Equatorial region and into the Northern Hemisphere. The BC carries salty and warm waters southward along the Brazilian coast and down to about 38°S, where it converges eastward with the Malvinas Current [31]. The MC originates from the Circumpolar Current that carries cold waters northward through the continental shelf of Argentina and Uruguay.

The northern portion of the South American continental shelf (0–5°S) is considerably large and is influenced by freshwater (100,000–220,000 m^3^ s^-1^) and sediment (11–13 x 10^8^ tons year^-1^) discharge from the Amazon River. The coastal region from about 2°S to 6°S has been described as semi-arid [32] because of its oligotrophic waters. The northeastern portion of the coast (5–15°S) is characterised by a markedly narrow continental shelf with a steep slope.

The coastal topography from about 16°S to about 20°S presents a broad shelf with the formation of the Royal Charlotte and Abrolhos Banks. The southern part of this region (up until 23°S) is characterised by a relatively narrow continental shelf. The region from Cabo Frio (23°S) to Cabo de Santa Marta Grande (28°40’S), the South Brazil Bight (SBB), presents a complex oceanographic profile with pronounced hydrographic variations and high productivity associated with upwelling regimes [33].

The region of Cabo Frio and its surroundings is influenced by the coastal upwelling of the deep South Atlantic Central Water (SACW), triggered by factors such as changes in coastal directions from north-south to east-west, near shore proximity of the 100 m isobath, and prevalence of north/northeast winds, especially during spring and summer [34,35]. The cold (~18°C), nutrient-rich waters of the SACW regularly influences the entire inner continental shelf of the SBB [33]. The continental shelf is broader from Cabo de Santa Marta Grande to the southern end of the Brazilian coast, and extends progressively southward to the edge of the continent, along the Uruguayan and Argentinean coasts [36].

### Ethics statement

The data collection approach, which included dead stranded animals, is under the protocol and regulations established by the Brazilian Stranding Network of Aquatic Mammals (REMAB) that is coordinated by the National Centre for Research and Conservation of Aquatic Mammals (CMA-ICMBio) from the Brazilian Ministry of the Environment (MMA) (http://www.icmbio.gov.br/cma). This study was approved by the Chico Mendes Institute for Biodiversity Conservation (ICMBio), and conducted under SISBIO license #32550–2. Both *Kogia* species are actually categorized as “Data Deficient” with undefined population trends on the IUCN Red List of Threatened Species (http://www.iucnredlist.org).

### Stranding events and environmental data

To study the stranding distribution patterns of the genus *Kogia* along the Brazilian coast, we compiled both published and unpublished information to produce a database of strandings from 1965 to 2014. The database includes data obtained from beach monitoring programmes along the Brazilian coast (IBAMA, 2001) and from literature survey (through an extensive search in Web of Science and Scopus). The data were carefully selected and only reliable information was considered. Specifically, we polled together information provided by specialists on marine mammals or data confirmed by a thorough taxonomical examination (e.g. skull examinations and photographs, S1 Table). Records with doubtful species identification or without satisfactory taxonomic material were excluded. When carcasses could not be identified on a species level but were recognized as belonging to the genus *Kogia* and biological tissue was available, we run a molecular procedure through the amplification of the segments of the cytochrome c oxidase I gene (COI) and the mitochondrial tRNAthr-control region (for details see Kocher et al. [37] and Ivanova et al. [38]). Sequence similarity and species identification based on COI and control region genes were obtained by comparison of similarity values to the sequences deposited at the GenBank using BLASTn (NCBI, available online). Additionally, to validate the reliability of the taxonomic identifications, we performed genetic analysis in selected samples from both species of *Kogia* that were previously recognized based on external or skull morphology. The collected information included date of event, location, sex, body length, evidence of human-related death (e.g. bycatch) [8].

We also compiled climatological data from a number of freely available environmental variables, which were used as predictors in the analysis of the stranding events. The environmental variables considered were: wind speed and wind direction from the Scatterometer Climatology of Ocean Winds (SCOW), chlorophyll-a and sea surface temperature from the Moderate Resolution Imaging Spectroradiometer (MODIS), and water depth from the 1 arc-minute global relief model (ETOPO1). To analyse the relationships of these environmental variables with the stranding events, we subdivided the Brazilian coast into 11 boxes, each 3° x 3° in size (1° equal to approximately 110 km) (see Fig 1). The environmental variables were spatially averaged in each box. In order to capture the environmental conditions that could best reflect the preferred habitat of these pelagic species [12,39–42], the positions of the boxes were chosen so that their central points would be as close as possible to the edge of the continental shelf (ECS).

The extreme northern region (0.5°S– 4°N) was not included in our statistical analyses due to lack of stranding events. Therefore, environmental variables from this area were not included in the dataset. Previous studies suggest that the absence of records in the region might be attributed to environmental forces other than those evaluated in this study, e.g. fluvial discharge [20,43,44].

Additionally, we collected the stomach content of a pregnant *K*. *breviceps* female found dead on December the 4th, 2005 in Saquarema (Rio de Janeiro State) in order to examine and identify remaining prey items with taxonomic relevance. In the stomach we found cephalopod beaks, which we used to identify the species and to estimate mantle lengths (mm) and weights (g), based on upper and lower rostral lengths (mm), following the method by Clarke [45], Santos [46], and Xavier and Cherel [47]. All the measurements were undertaken using a stereomicroscope with ocular micrometer and precision of 0.1 mm. The relative importance of prey taxa for the diet of the specimen assessed was estimated by calculating the percentage of a prey relative to each species number (FN%) in relation to the total number of prey consumed [48]. Moreover, in order to evaluate the diet composition and ecological aspects of these species, we surveyed the literature for information concernig the stomach content of *Kogia* found in Brazilian waters.

### Statistical analyses

In order to explore the spatial distribution of the stranding events and examine for trends or dissimilarities between the two species along the Brazilian coast, a synoptic exploratory map was produced using the software QGIS (version 2.6.1. Brighton).

A Principal Component Analysis (PCA) was then applied as a preliminary investigation for potential correlations between stranding events and environmental data. PCA is one of the most frequently used multivariate statistical techniques, which consists in an orthogonal transformation of a set of observations of possibly correlated variables into a set of linearly uncorrelated variables called principal components, PCs [49]. The magnitude of each eigenvalues is interpreted as the amount of variability explained by the first PCs. With this multivariate analysis one can quantify the effect of each variable on each PC, which is known as the loading or eigenvector. The loadings provide an indication of the magnitude and sign of the correlation (i.e. positive or negative) between the original variables and the specific principal component.

To further explore associations among variables, we performed a multiple regression analysis using the Generalized Linear Model (GLM) method to evaluate the changes in the number of stranding events with respect to the selected environmental variables: wind speed (Wspe), wind direction (Wdir), chlorophyll-a (Chl-*a*), sea surface temperature (SST), and water depth (Depth). Preliminary results of the GLM regression based on a Poisson distribution showed an overdispersion of the data when running the analyses for the *K*. *sima* (KS) and for the total number of stranding events (TN). Therefore, the models were fitted considering a negative binomial distribution with a log link function. For *K*. *breviceps* (KB) the models were built based on a Poisson distribution. We first analysed the relationship between numbers of stranding (for each *Kogia* species, respectively, KS and KB, and also for both species combined, TN) and each separated environmental variable. In a second step, we evaluated which combination of environmental variables best described the observed number of strandings by fitting different probabilistic models and by selecting the preferred one using the Akaike’s Information Criterion (AIC). The AIC method provides a measure of the quality of a statistical model by balancing goodness of fit versus model complexity [50]. According to this criterion the preferred model is the one that has the lowest AIC value.

All the statistical analyses were carried out using R (The R Foundation for Statistical Computing, Viennna, Austria, v.3.1.3) and with the package ‘MASS’.

## Results

In the time period from 1965 to 2014, 100 stranding events of the genus *Kogia* were recorded along the Brazilian coast. From these occurrences, 58 involved the species *K*. *sima* and 42 the species *K*. *breviceps*. The stranding events covered a coastal region from 2°S to 33°S, and were especially abundant in the southern- and northern-most parts of this region (Fig 1). No *Kogia* stranding events have been recorded to date in the extreme northern region from 0° to 4°N), although an extensive dedicated survey has been carried out there over the last seven years (Fig 1). The strandings of KB were concentrated around the southern portion of the Brazilian coast, while KS carcasses were distributed more homogeneously (Fig 1). The four southernmost boxes (08–11, Fig 1) contained 66.7% (n = 28) of the total stranding records of KB, while KS stranding events were comparatively less frequent in this area, accounting for 37.9% (n = 31) of the total stranding records of this species. Furthermore, 31 stranding events of KS, 53.4% of the total of this species, occurred in the four northernmost boxes (04–01, Fig 1).

The temporal variability of the stranding events was highly variable. For KS, a peak was clearly identified in the austral summer (January and February) and austral winter (June and July), while for KB peaks occurred in January, May and September (Fig 1).

**Fig 2:**
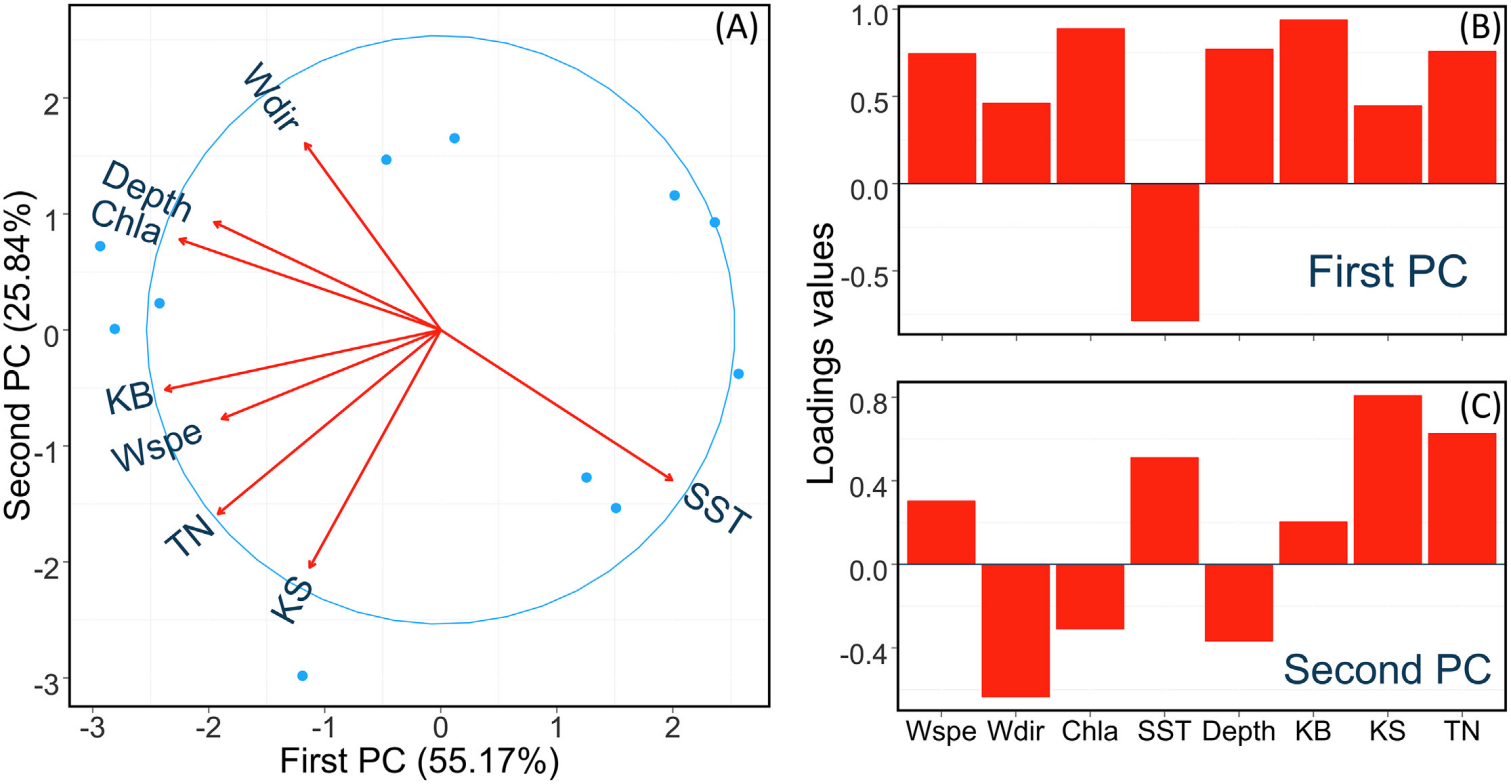
Principal Component Analysis. Panel A shows the ordination biplot. The arrows indicate the loadings on the respective PCs. The length of the arrows indicates the power of the relationship and the angle between two arrows provides the degree of correlation between the corresponding variables. Panels B and C show barplots of the loadings illustrating the sign and the strength of the relationships between variables and first (B) and second (C) PCs.

The PCA analysis showed that the first two principal components (PCs) explain over 80% of the total variance, with first PC explaining 55.1% and second PC explaining 25.84% (Fig 2). The loadings of each variable revealed that KB, TN, Chl-a, Depth, Wspe, and SST are strongly correlated with the first PC (loading values higher than 0.7), with SST being the only variable showing a negative correlation with PC1 (Fig 2). KS, TN, and Wdir are correlated with the second PC (loading values higher than 0.6), with Wdir showing a negative correlation with the second PC, although this component does not explain much of the variance.

**Fig 3:**
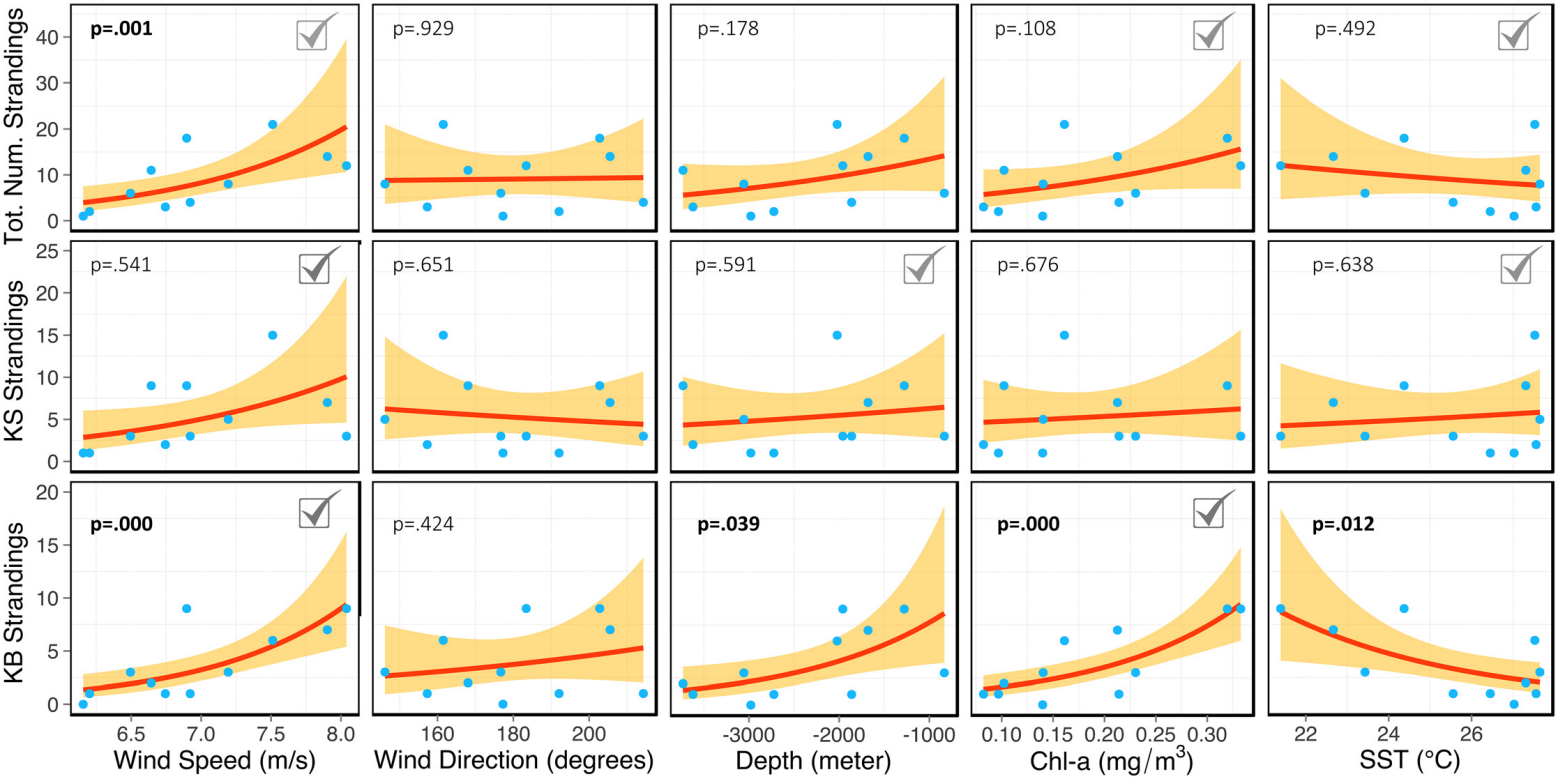
Probabilistic model fits. Each panel includes stranding data (blue dots), probabilistic fits based on Generalized Linear Models (red lines), 95% confidence intervals of the fit (shadowed yellow areas) respective p-values (significance at p < 0.05, showed in boldface). The check mark symbols indicate the relevant variables of the selected predictive GLM models for *K*. *breviceps* (KB), *K*. *sima* (KS) and for total number of strandings (TN, both species combined).

The effects of each environmental variable on the stranding events were investigated using GLM regression. First, we assessed the effects of each environmental variable on stranding events (S2 Table and Fig 3). Both TN and KB showed a positive and significant relationship with Wspe (p < 0.01). The same relationship was observed between KS and Wspe, although with less significance (p = 0.0541). No further significant associations were observed for TN and KS. However, most variables, except Wdir, were significantly associated with KB. These associations with KB were positive for Chl-a, Depth, and Wspe, and negative for SST. These results indicate that the stranding events of KB are more likely to occur in coastal regions adjacent to highly productive and temperate waters with high wind intensity and broader continental shelves (i.e. shallower areas; Fig 1). Second, we examined the combined effects of the environmental variables on the stranding events by running a series of additive GLM models (Table 1 and S3 Table). The preferred GLM models for TN and KB were similar; both sharing a positive and significant relationship with Wspe and Chl-a, but the preferred model for TN also included SST as a predictor. According to the AIC, the preferred model for predicting the stranding events of KS included a positive association with Wspe, SST and Depth (Table 1). However, regression with Depth produced a considerable small slope and was not statistically significant, indicating a low predictive power of this variable on KS strandings. Wind direction resulted insignificant both in the single and in the combined effect models. Being relevant in all preferred models, wind speed is an important predictor of the stranding events of both species (Table 1). Furthermore, KB carcasses are more likely found in regions adjacent to waters with relatively high Chl-a, whereas KS carcasses are more likely found in regions adjacent to relatively warmer waters. Subsequent important models, i.e. models with very close AIC scores, suggest that Wspe alone is a good predictor for TN (S3 Table). For KS the second best model includes Chl-a and SST but not Depth, and the third best considers only Chl-a and SST (S3 Table). For KB the second most relevant model, includes Depth. The GLM modelling indicates that single models, i.e. models that included each predictor variable separately, were not sufficient to predict the variation in the stranding data.

**Table 1:** Summary statistics for preferred model fits. A series of models with associated Akaike’s Information Criterion (AIC) indices are reported along with parameters estimates for intercept “α” and slope “β”, their respective standard error, and p-values (* indicates p < 0.05).

Species	AIC	Parameter	Estimate ± SE	p-value
**TN**	69.709	α	-10.9993 ± 4.6259	0.0174*
		βWspe	0.8795 ± 0.3004	0.0034*
		βChl-a	6.1791 ± 3.1823	0.0521
		βSST	0.2255 ± 0.1221	0.0648
**KB**	45.666	α	-4.1109 ± 1.9254	0.0328*
		βWspe	0.6108 ± 0.2916	0.0362*
		βChl-a	5.0874 ± 2.1536	0.0182*
**KS**	61.336	α	-1.0270 ± 3.9640	0.0096*
		βWspe	0.8632 ± 0.3226	0.0074*
		βSST	0.2622 ± 0.1076	0.0148*
		βdepth	0.00039 ± 0.00026	0.1322

At least 56 prey specimens of cephalopods were recovered from the stomach content of a pregnant KB female stranded in Saquarema, Rio de Janeiro state (Table 2). We found that *Megalocranchia maxima* was the most representative prey item (n = 12; FN% = 21.4), followed by a species from the genus *Chiroteuthis* (n = 7; FN% = 12.5). These two cephalopod species, in addition to unidentified items (n = 10) of the suborder *Oegopsina*, represented 51.7% of the total food content. We also found five specimens of *Ancistrocheirus lesueuri*, *Histioteuthis corona corona* and an unidentified species of the genus *Histioteuthis*. The most important prey species in terms of biomass was *Histioteuthis corona corona*, with an estimated mean weight of 673 g (range: 357.2–1150.1 g), and *Ancistrocheirus lesueuri* with an estimated mean weight of 356 g (range: 333.5–379.6 g).

**Table 2:** Food items recovered from the stomach of a stranded pygmy sperm whale (*K*. *breviceps*). FN% = (number of preys of one species x 100) / (number of preys of all species); ML = mantle length; WT = weight.

Family	Species	(N)	ML (mm)		WT (g)		FN%
			mean	range	mean	range	
**Ancistrocheiridae**	*Ancistrocheirus lesueuri*	5	265.4	261.4–269.5	356.5	333.5–379.6	8.93
**Histioteuthidae**	*Histioteuthis corona corona*	5	129.9	109.3–158.0	673	357.2–1150.1	8,93
	*Histioteuthis macrohista*	2	97.5	--	202.7	--	3.57
	*Histioteuthis* sp.	5	38.4	30.8–39.7	36.7	24.4–67.2	8.93
**Ommastrephidae**	*Ornithoteuthis antillarum*	3	144.8	131.9–156.6	38.8	31.2–46.1	5.36
	*Illex argentinus*	1	343.6	--	735.9	--	1.78
**Enoploteuthidae**	*Abralia* sp.	1	--	--	--	--	1.78
	*Enoploteuthis* sp.	2	--	--	--	--	3.57
**Cranchiidae**	*Megalocranchia maxima*	12	234.0	106.2–283.4	61.4	12.3–81.9	21.42
	*Taonius* sp.	1	--	--	--	---	1.78
**Chiroteuthidae**	*Chiroteuthis* sp.	7	103.9	94.6–109.2	28.8	21.4–33.2	12.50
**Octopoteuthidae**	*Octopoteuthis* sp.	2	116.6	110.5–122.6	97.6	86.0–109.3	3.57
**Oegopsina**	Oegopsina unidentified	10	--	--	--	--	17.86

## Discussion

The relatively common and wide-ranging occurrence of *Kogia* strandings provides a unique opportunity to increase knowledge of these species that are difficult to study at sea [2,4,5,51]. A comparative study carried out in Hawaii, for example, demonstrated that *Kogia* whales have significantly higher frequency of stranding when compared to free-ranging observation at sea [52]. Overall, our results suggest that *Kogia* species are relatively common in Brazilian waters, despite the rare sightings at sea. The spatial distribution of strandings appears to be rather heterogeneous throughout the Southwest Atlantic coast. No specimen of the genus *Kogia* has been found stranded in the northernmost regions of Brazil, French Guiana, Trinidad and Tobago, Suriname, and Guyana [20,53]. However, a few records have been reported in the Venezuelan coast [54]. The absence of *Kogia* data for the northern region of Brazil has been associated to the fresh water and sediment discharge from the Amazon and Orinoco rivers, which might prevent drifting carcasses to strand by pushing them offshore [20]. Furthermore, the river outflow combined with the broad continental shelf of the Amazon region might impede living *Kogia* to approach the coast and thus reducing the probability of stranding. The Amazon River, in fact, produces a mean average fluvial discharge of 175,000 m^3^ s^-1^, which has an important effect on the marine biodiversity of that region [55]. Studies on the biogeography of pelagic dolphins (e.g. genus *Stenella* and *Delphinus* sp.) reported data with similar gaps [43,44,56], thus lending further weight to the possibility that stranding of cetaceans are less likely to occur in the Amazon coastal region. Siciliano et al. [53] compiled stranding data of aquatic mammals for the northern coast of Brazil and reported a high prevalence of inshore dolphins (e.g. *Sotalia guianensis*) in this region.

Both *Kogia* species have overlapping habitats, but some latitudinal discrepancy in their distribution has been previously reported [5]. The significant correlations between *K*. *breviceps* and high chlorophyll-a concentration and, to a less extent, lower sea surface temperature, corroborate the knowledge that this species exhibits a preference for productive subtropical and temperate waters [2,4,5]. This is consistent with the fact that the highest frequency of strandings occurred in the southern portion of the studied region, which is associated with the Brazil-Malvinas Confluence (BMC) and the upwelling systems of Cabo Frio and South Brazil Bight (Figs 1 and 3) [57–59]. Moreover, the broader continental shelf in southern Brazil is a potential explanation for the observed significant relationship between lower depth and number of strandings of *K*. *breviceps*, although this was only detected by the single GLM model approach (Figs 1 and 3). Contrasting results were found for *K*. *sima*, which strandings were associated to higher sea surface temperature and showed no significant correlation with chlorophyll-a, thus supporting the preference of this species for a more tropical habitat (Table 1). This conclusion is based on the higher number of strandings of *K*. *sima* in the northern regions (Fig 1), which are characterized by warm and low productivity waters [32]. These results are in line with the current knowledge about habitat preferences of these species [4,5,11,12,30]. Also consistent to previous studies, some *K*. *sima* carcasses have been found stranded at the southern limit of the Brazilian coast, but only stranding of *K*. *breviceps* have been reported in more temperate waters, further south along the Southwest American coast, including the coasts of Uruguay and Argentina [20,60]. Our results also show that the environmental variable most strongly correlated with stranding events is wind speed (Figs 2 and 3, Table 1). This is not surprising given the important effect that wind speed can have on drifting carcasses and probably on debilitated whales, given that a considerable proportion of the strandings involved impaired living individuals of *K*. *breviceps* (19.1%) and *K*. *sima* (22.4%). High incidence of live strandings are commonly described for some regions, such as South Africa (KB: 32.6%; KS: 26.2%) [61] Atlantic and Gulf coast of the U.S. (KB = 33.0%) [62] and New Zealand (KB = 13.0%) [63].

Our analyses on the feeding ecology of *Kogia* species in Brazilian waters are consistent with other studies suggesting deep sea feeding habits and a predominant preference for oceanic cephalopods, although fish and crustaceans are also part of the diet of these cetaceans [2,23,64–66]. The cephalopod families Histioteuthidae and Cranchiidae constituted the main prey species in the diet of the stranded *K*. *breviceps*. The relevance of these organisms for the dietary composition of *K*. *breviceps* and *K*. *sima*, have been confirmed by several studies carried out in different regions of the world, including Hawaii [66], Northeast Atlantic [64] and U.S. mid-Atlantic coast [67]. The cephalopods prey species we found in the stranded individual have a large distributional range and occupy both mesopelagic and bathypelagic ocean zones. This is consistent with literature data from the Brazilian coast (summarised in Table 3) indicating similar diets for both *Kogia* species. Recently, also a study carried out in the U.S. mid-Atlantic [67] suggested that both species present similar feeding ecologies and occupy equivalent trophic niches. However, our regression analysis shows that environmental variables such as chlorophyll and sea surface temperature may have a dissimilar influence on the latitudinal distribution of the two species, pointing to different feeding habits.

**Table 3:** Literature information concerning cephalopod prey species recovered in stomach contents of *K*. *sima* and *K*. *breviceps* stranded along the Brazilian coast.

Family of prey species	Prey species	*K*. *sima*	*K*. *breviceps*	References
**Sepiolidae**	*Semirossia tenera*	Y	-	[23]
	*Heteroteuthis dispar*	Y	Y	[23]
	*Heteroteuthis atlantis*	Y	Y	[23,68]
**Lycoteuthidae**	*Lycoteuthis lorigera*	Y	Y	[23]
	*Lycoteuthis diadema*	-	Y	[69]
**Enoploteuthidae**	*Abralia redfieldi*	Y	-	[23]
	*Abralia veranyi*	-	Y	[24]
	*Abralia* sp.	-	Y	[TS]
	*Enoploteuthis* sp.	-	Y	[69, TS]
**Octopoteuthidae**	*Octopoteuthis* sp.	Y	Y	[30,70,71, TS]
**Onychoteuthidae**	*Moroteuthis ingens*	Y	-	[23]
	*Moroteuthis robsoni*	Y	Y	[23]
	*Onychoteuthis banksii*	-	Y	[70]
**Histiotheuthidae**	*Histioteuthis corona corona*	-	Y	[TS]
	*Histioteuthis macrohista*	-	Y	[TS]
	*Histioteeuthis* spp.	Y	Y	[30,67,70,71, TS]
**Ommastrephidae**	*Illex argentinus*	Y	Y	[30,68,71, TS]
	*Ornithoteuthis antillarum*	Y	Y	[30, TS]
	*Ommastrephes bartramii*	-	Y	[71]
**Chiroteuthidae**	*Chiroteuthis veranyi*	Y	Y	[23,71,72]
	*Chiroteuthis* sp.	Y	Y	[TS]
**Cranchiidae**	Unknown	Y	-	[23]
	*Megalocranchia maxima*	-	Y	[TS]
	*Liocranchia reinhardti*	-	Y	[24]
	*Taonius* sp.	-	Y	[TS]
**Bolitaenidae**	*Japetella diaphana*	Y	-	[23,72]
**Mastigoteuthidae**	*Mastigoteuthis* sp.	-	Y	[70]
**Alloposidae**	*Haliphron atlanticus*	-	Y	[24]
**Neoteuthidae**	*Neoteuthis thielei*	-	Y	[70]
**Ancistrocheiridae**	*Ancistrocheirus lesueurii*	-	Y	[TS]

TS = This Study; Y = Yes.

## Conclusions

We investigated the stranding patterns of two rare *Kogia* species based on a number of predictive enironmental variables. *K*. *sima* shows a preference for warm waters of the northeastern Brazilian coast, whereas, *K*. *breviceps* exhibts a preference for temperate and productive waters. The most important environmental predictor explaining the stranding events is wind speed. This variable, however, may have a strong postmorten effect on drifting carcasses and on debilitated whales. Consistently with previour finding, our results on feeding ecology indicates a preference of *Kogia* species for oceanic cephalopods. Additionally, the lack of stranding events in the Amazon coast suggest that strong river outflow and discharge might prevent drifting carcasses to strand by pushing them offshore. By using the available information as a qualitative proxy for habitat preference and trophic ecology in association to environmental variables, our work provides a comprehensive assessment of *Kogia* stranding data along the Brazilian coast. Our findings also highlights the scientific value of stranding monitoring programmes to better understand the impacts of environmental conditions on marine mammals and their vulnerabilities to changes.

## Supporting Information

S1 Table Stranding data of genus *Kogia* along the Brazilian coast. KS = *Kogia sima*; KB = *Kogia breviceps*.
(DOCX)

S2 Table Summary statistics of the Generalized Linear Model method for the single effect of each environmental variable on *Kogia* strandings. Reported are parameter estimates (± standard error) for intercept “α” and slope “β” of each model. Corresponding p-values are p < 0.05 for *, p < 0.01 for **, and p < 0.001 for ***.
(DOCX)

S3 Table Model selection from a series of additive models. The selection is based on the Akaike’s Information Criterion (AIC) and Akaike’s Weights (W_AIC_). The best models are the one that have the lowest AIC and highest W_AIC_, here highlighted in bold and with superscript (1). The second and third subsequent most important models according to AIC are indicated with superscripts (2) and (3), respectively.
(DOCX)
