# Supplementary material for: Stranding Events of Kogia Whales along the Brazilian Coast
Source: PLoS One. 2016 Jan 5;11(1):e0146108. doi: 10.1371/journal.pone.0146108 (PMC4701718; doi:10.1371/journal.pone.0146108)
Supplement: S1 Table — KS = Kogia sima; KB = Kogia breviceps. (DOCX) [file pone.0146108.s001.docx]

**S1 Table. Stranding data of genus *Kogia* along the Brazilian coast. KS = *Kogia sima*; KB = *Kogia breviceps*.**

| **Species** | **Localities** | **Month** | **Year** | **Collection** | **Sources** | **Lat** | **Long** |
| --- | --- | --- | --- | --- | --- | --- | --- |
| KS | Praia do Mar Grosso, RS | Jul | 1983 | MORG 495 | [1] | -31,93050 | -51,84900 |
| KS | Rio Grande do Sul, RS | Oct | 1986 | -- | TS | -32,32362 | -52,28395 |
| KS | Itaparica, BA | Jan | 1990 | -- | [2] | -12,91748 | -38,62800 |
| KS | Praia de Jericoacoara, CE | Feb | 1992 | -- | [3] | -2,78867 | -40,48966 |
| KS | Ceará State, CE | Jul | 1992 | -- | [4] | -3,992059 | -38,23905 |
| KS | João Pessoa, PB | Mar | 1994 | UFPB 2021 | [5] | -7,16592 | -34,79578 |
| KS | Praia das Monçoes, PR | Apr | 1994 | MCEM 108 | [6] | -25,72333 | -48,48353 |
| KS | Curitiba, PR | -- | 1994 | -- | [4] | -25,71867 | -48,48064 |
| KS | João Pessoa, PB | Jan | 1995 | UFPB 2022 | [1] | -7,12405 | -34,82314 |
| KS | Entre Rios, BA | Jun | 1995 | UFBA/CET 018 | [7] | -12,30663 | -37.82408 |
| KS | Praia do Pinhal, RS | Jul | 1995 | MOVI 5298 | [8] | -30,26139 | -50,23512 |
| KS | Praia de Itapoá, SC | Jan | 1996 | MOVI 5587 | [4] | -26,11656 | -48,60011 |
| KS | Rio Tinto, PB | Jan | 1996 | UFPB 2405 | [9] | -6,779302 | -34,91801 |
| KS | Praia de Pitimbu, PB | Jun | 1997 | -- | [10] | -7,471569 | -34,80416 |
| KS | Praia Grande, SP | Feb | 1998 | CEEMAM 20 | [3] | -24,08531 | -46,59501 |
| KS | Ceará State, CE | Jul | 1998 | -- | TS | -4,503196 | -37,72466 |
| KS | Mata de São João, BA | Apr | 1999 | CCPM 0050 | [11] | -12,59381 | -38,02750 |
| KS | Pirambú, SE | Jul | 1999 | CCPM 0056 | [12] | -10,86838 | -36,96774 |
| KS | Rio Grande, RS | Oct | 1999 | -- | [11] | -32,26573 | -52,24057 |
| KS | Salvador, BA | May | 2000 | CCPM 0081 | [11] | -16,06903 | -38,94299 |
| KS | Vera Cruz, BA | Jun | 2000 | CCPM 0083 | [13] | -17,22483 | -39,21442 |
| KS | Saquarema, RJ | Dec | 2000 | UERJ-MQ 144 | [14] | -22,93537 | -42,62500 |
| KS | Peruíbe, SP | -- | 2000 | -- | [15] | -24,14685 | -46,71526 |
| KS | Praia de Serrambi, PE | Jan | 2001 | -- | TS | -8,56299 | -35,00767 |
| KS | Ceará State, CE | Feb | 2001 | -- | [3] | -3,92377 | -38,32352 |
| KS | Vera Cruz, BA | Feb | 2001 | CCPM 0103 | TS | -13,0976 | -38,75069 |
| KS | Campos dos Goytacazes, RJ | Jun | 2001 | MCNM 195, | [16] | -22,06636 | -41,09187 |
| KS | Between Torres and Chuí, RS | -- | 2001 | -- | [17] | -31,42955 | -51,11222 |
| KS | São Sebastião, SP | Jun | 2002 | SOSMM 097 | [10] | -23,76821 | -45,73550 |
| KS | Lauro de Freitas, BA | Sep | 2004 | CCPM 0204 | [18] | -13,01132 | -38,50447 |
| KS | Ceará State, CE | Jan | 2005 | -- | [3] | -2,81388 | -40,24649 |
| KS | Salvador, BA | Feb | 2005 | CCPM 0209 | [19] | -12,99219 | -38,43844 |
| KS | Salvador, BA | Mar | 2005 | CRMA 0014 | [18] | -12,94054 | -38,33082 |
| KS | Rio de Janeiro, RJ | Jun | 2005 | UERJ-MQ 206 | [13] | -23,03135 | -43,47610 |
| KS | Itanhaém, SP | Dec | 2007 | CEEMAM 332 | [10] | -24,18442 | -46,78312 |
| KS | Imbé, RS | Oct | 2008 | GEMARS 1311 | TS | -29,95888 | -50,11113 |
| KS | Maranhão, MA | Feb | 2009 | -- | [11] | -2,34008 | -43,31306 |
| KS | Barroquinha, CE | Feb | 2009 | AQ-02C0511/334 | TS | -2,88555 | -41,26416 |
| KS | Cabo Frio, RJ | Apr | 2009 | GEMM 165 | TS | -22,68303 | -41,99539 |
| KS | Itaparica, BA | May | 2009 | -- | TS | -13,09085 | -38,73860 |
| KS | Guarujá, SP | Jul | 2009 | C.05.1.2.114 09 | [10] | -24,00084 | -46,26228 |
| KS | Aquiraz, CE | Aug | 2009 | AQ-02C0510/350 | TS | -3,83976 | -38,39200 |
| KS | Cidreira, RS | Jul | 2010 | GEMARS 1407 | TS | -30,19982 | -50,21109 |
| KS | Palmares do Sul, RS | Nov | 2010 | GEMARS 1421 | TS | -30,55698 | -50,37115 |
| KS | Ilha Grande, RJ | Jan | 2011 | -- | TS | -23,19125 | -44,25212 |
| KS | Macaé, RJ | Sep | 2011 | GEMM 333 | TS | -22,31465 | -41,71012 |
| KS | Linhares, ES | Oct | 2011 | -- | TS | -19,82655 | -40,05811 |
| KS | Ceará State, CE | Jan | 2012 | -- | TS | -4,38192 | -37,84997 |
| KS | Amontada, CE | Aug | 2012 | AQ-02C0511/556 | TS | -3,01863 | -39,66275 |
| KS | Beberibe, CE | Jan | 2012 | AQ-02C0512/528 | TS | -4,39467 | -37,82696 |
| KS | Rio Grande, RS | ND | 1996 | -- | [7] | -30,87594 | -50,60330 |
| KS | Fortaleza, CE | Mar | 2013 | AQ-02C0511/580 | TS | -3,70561 | -38,55821 |
| KS | Fortaleza, CE | Mar | 2014 | AQ-02C0512/582 | TS | -4,39883 | -37,79963 |
| KS | Fortaleza, CE | Jun | 2013 | AQ-02C0512/585 | TS | -3,69319 | -38,58530 |
| KS | Fortaleza, CE | Jun | 2013 | AQ-02C0512/586 | TS | -3,69319 | -38,58530 |
| KS | Luís Correria, PI | Jan | 2012 | -- | TS | -2,91043 | -41,53348 |
| KS | Palmares do Sul, RS | Jan | 2014 | -- | TS | -30,44266 | -50,30465 |
| KS | Maceió, AL | Jan | 2014 | -- | TS | -9,52942 | -35,59207 |
| KB | Santos, SP | Oct | 1965 | DZ 10597 | [20] | -23,96935 | -46,34744 |
| KB | Arraial do Cabo, RJ | Dec | 1983 | MZ-USP 19482 | [21] | -22,94809 | -42,14141 |
| KB | Praia do Cassino, RS | Sep | 1986 | MORG 891 | [22] | -32,18675 | -52,15208 |
| KB | Fernando de Noronha Is., PE | May | 1987 | -- | [23] | -3,84855 | -32,43588 |
| KB | Tramandaí, RS | Sep | 1988 | -- | [24] | -30,00767 | -50,13206 |
| KB | Cananéia, SP | Jan | 1988 | -- | [25] | -25,20417 | -47,98077 |
| KB | Mar Grosso, RS | May | 1989 | MORG 2014 | [26] | -31,99231 | -51,92706 |
| KB | Mar Grosso, RS | May | 1989 | -- | [26] | -31,85839 | -51,72750 |
| KB | São José do Norte, RS | Sep | 1994 | MCN 2561 | [27] | -32,13095 | -52,06735 |
| KB | Rio Grande, RS | Apr | 1995 | -- | TS | -33,43021 | -52,95999 |
| KB | Ilha Comprida, SP | Sep | 1995 | -- | [28] | -24,80932 | -47,63932 |
| KB | Praia de Piçarras, SC | Jan | 1997 | MOVI 7536 | [29] | -26,75990 | -48,67322 |
| KB | Rio Tinto, PB | Oct | 1997 | UFPB 2558 | [24] | -6,893453 | -34,87686 |
| KB | Between Torres and Chuí, RS | -- | 1997 | -- | [30] | -31,43098 | -51,10726 |
| KB | Ceará State, CE | Mar | 1999 | -- | [3] | -3,71811 | -38,46249 |
| KB | Praia Grande, SP | Jul | 2000 | -- | [31] | -24,01923 | -46,43719 |
| KB | Santos, SP | Jul | 2000 | -- | TS | -23,97126 | -46,33260 |
| KB | Between Torres and Chuí, RS | -- | 2001 | -- | [17] | -31,43098 | -51,10726 |
| KB | Barra de Maxaranguape, RN | -- | 2003 | -- | [32] | -5,51319 | -35,25875 |
| KB | Valença, BA | Feb | 2004 | CCPM 0182 | [18] | -13,29082 | -38,96349 |
| KB | Arraial do Cabo, RJ | Sep | 2005 | GEMM 090 | TS | -22,97532 | -42,03568 |
| KB | Peruibe, SP | Sep | 2005 | -- | [10] | -24,32320 | -46,99304 |
| KB | Espírito Santo, ES | Nov | 2005 | -- | [33] | -18,74146 | -39,74640 |
| KB | Saquarema, RJ | Dec | 2005 | GEMM 093 | TS | -22,93612 | -42,47955 |
| KB | Cascavel, CE | Jan | 2006 | AQ-02C0512/191 | TS | -4,04066 | -38,19219 |
| KB | Rio Grande do Sul, RS | Jul | 2006 | -- | TS | -32,50988 | -52,38065 |
| KB | Aracati, CE | Sep | 2007 | AQ-02C0521/297 | TS | -4,55289 | -37,67090 |
| KB | Ceará State, CE | Feb | 2009 | -- | TS | -2,88648 | -41,26731 |
| KB | Ilha de Vera Cruz, BA | May | 2009 | CRMA 0032 | [34] | -12,95038 | -38,60683 |
| KB | Rio Grande do Sul, RS | Sep | 2009 | -- | TS | -33,14098 | -52,65078 |
| KB | Amontada, CE | Nov | 2009 | AQ-02C0520/362 | TS | -2,97000 | -39,75833 |
| KB | Rio Grande do Sul, RS | Nov | 2009 | -- | TS | -31,48055 | -51,16322 |
| KB | Macaé, RJ | May | 2010 | GEMM 192 | TS | -22,30777 | -41,70085 |
| KB | Praia Grande, SC | Jan | 2011 | -- | [35] | -29,25692 | -49,66121 |
| KB | Praia de Itaguaçu, SC | Aug | 2011 | -- | [35] | -26,18253 | -48,52725 |
| KB | Rio Grande do Sul, RS | Jan | 2012 | -- | TS | -33,31823 | -52,81987 |
| KB | Mostardas, RS | Apr | 2012 | -- | TS | -30,85476 | -50,59009 |
| KB | Mostardas, RS | May | 2012 | GEMARS 1496 | TS | -31,11134 | -50,76965 |
| KB | Itapipoca, CE | May | 2012 | AQ-02C0521/544 | TS | -3,14425 | -39,46105 |
| KB | Praia de Itapoá, SC | Nov | 2012 | -- | [35] | -26,11694 | -48,60052 |
| KB | Tamandaré, PE | Aug | 2013 | -- | TS | -8,73705 | -35,08744 |
| KB | Rio Grande do Sul, RS | Apr | 2014 | -- | TS | -32,59528 | -52,41263 |

TS = This study.

**Abbreviations and full names of the scientific collections and Museums mentioned in the S1 Table.**

| CCPM / CRMA | Coleção Científica do Projeto Mamíferos Marinhos — PROMAMA, Bahia |
| --- | --- |
| CEEMAM | Centro de Estudos de Encalhes de Mamíferos Marinhos, São Paulo |
| DZ | Departamento de Zoología, Secretaría de Agricultura, São PauJo |
| GEMM | Grupo de Estudos de Mamíferos Marinhos da Região dos Lagos, Rio de Janeiro |
| MCEM | Museu do Centro de Estudos do Mar, Universidade Federal do Paraná, Paraná |
| MCNM | Coleção Mastozoológica do Museu de Ciências Naturais, Rio Grande do Sul |
| MCN | Museu de Ciências Naturais, Fundação Zoobotânica do RS, Rio Grande do Sul |
| MORG | Museu Ocenográfico ‘Eliézer de Carvalho Rios’ — FURG, Rio Grande do Sul |
| MOVI | Museu Oceanográfico do Vale do Itajaí, Itajaí, Santa Catarina |
| MZ-USP | Museu de Zoologia da Universidade de São Paulo, São Paulo |
| SOSMM | SOS Mamíferos Marinhos – Instituto Terra & Mar, São Paulo |
| UFPB | Universidade Federal da Paraíba, Paraíba |
| UERJ-MQ | Projeto Mamíferos Aquáticos — UERJ, Rio de Janeiro |
| UFBA | Universidade Federal da Bahia, Bahia |
| AQ-AQUASIS | Associação de Pesquisa e Preservação de Ecossistemas Aquáticos – AQUASIS, Ceará |
| GEMARS | Grupo de Estudos de Mamíferos Aquáticos do Rio Grande do Sul, Rio Grande do Sul |

**S1 Table References**

1. Sampaio, C. L. S., Aroucha, E. C. (2000). Registro de cachalote anão, *Kogia Simus* (Owen, 1866) no litoral da Bahia, nordeste do Brasil. *Bioikos 14*: 28-33.

2. Alves, T. T. J., Ávila, F. J. C., Oliveira, J. A., Furtado-Neto, M. M. A., Monteiro-Neto, C. (1996). Registros de cetáceos para o litoral do estado do Ceará, Brasil. *Arq. Cienc. Mar.* 30, 79-92.

3. Meirelles, A. C. O., Monteiro-Neto, C., Martins, A. M. A., Costa, A. F., Barros, H. M. D. R., and Alves, M. D. O. (2009). Cetacean strandings on the coast of Ceará, north-eastern Brazil (1992–2005). *J. Mar. Biol. Assoc. United Kingdom* 89, 1083-1090.

4. Lucena, A., Paludo, D., Langguth, A. (1998). New records of Odontoceti (Cetacea) from the coast of Paraíba, Brazil. *Rev. Nordest. Biol. 12*, 19-27.

5. Zanellato, R. C., Guierra, C.M. Primeiro registro de cachalote anão *Kogia simus* Owen, 1866 (Cetacea, Physeteridae) para a costa do Paraná, Brasil. *In VI Reunião de Trabalhos de Especialistas em Mamíferos Aquáticos da América do Sul*; 1994 Oct 24-28; Santa Catarina, Brazil. p. 109-110

6. Santos, R. A., and Haimovici, M. (2001). Cephalopods in the diet of marine mammals stranded or incidentally caught along southeastern and southern Brazil (21-34°S). *Fish. Res.* 52, 99–112.

7. Soto, J. M., Caseca-Santos, L. R., Ternes-Silva, S. Dados sobre a captura acidental de cetáceos pela frota pesqueira de Itajaí (Santa Catarina, Brasil), através de redes de emalhe de superfície. In *VII Reunión de Trabajo de Especialistas en Mamíferos Acuáticos de América del Sur;* 1996 Oct 22-25; Viña del Mar, Chile. p. 63.

8. Zanellato, R. C., Rosas, F. C. W., and Santos, R. A. Análise do conteúdo estomacal de um cachalote anão *Kogia simus* (Cetacea: Kogiidae), registrado para o litoral do Paraná, Brasil. In *VII Reunión de Trabajo de Especialistas en Mamíferos Acuáticos de América del Sur*; 1996 Oct 22-25; Viña del Mar, Chile. p. 82.

9. Marques, C. C., El-Deir, A. C. A., Getúlio, R. F., Andrade, C. E. R., Costa, G. M., Lessa, R. P. T., Hellebrandt, D. Registro de captura acidental de um cachalote-anão, *Kogia simus* (Owen, 1866) (Cetacea, Odontoceti) ao largo da costa da Paraíba. *In XXII Congresso Braileiro de Zoologia*; 1998 Feb 08-13; Pernambuco, Brazil. p. 334.

10. Santos, M. C. O., Siciliano, S., de Castro Vicente, A. F., Alvarenga, F. S., Zampirolli, É., de Souza, S. P., and Maranho, A. (2010). Cetacean records along São Paulo state coast, Southeastern Brazil. *Brazilian J. Oceanogr.* 58, 123–142.

11. Maia-Nogueira, R., Baracho, C. G., Serra, S. D. (2001). Revisão dos Registros do gênero *Kogia* (GRAY, 1846) (Cetacea, Physeteridae, Kogiinae) no litoral do nordeste do Brasil, incluindo dados osteológicos. *Bioikos 15*, 50-59.

12. Menezes, R.B. (2005) *Encalhes de cetáceos (Ordem Cetacea), entre 1993 e 2004, no litoral do Rio Grande do Sul-RS (Thesis)*. Rio Grande do Sul: FURG - Fundação Universidade Federal do Rio Grande.

13. Dorneles, P. R., Lailson-Brito, J. J., Azevedo, A. F., Fragoso, A. B. L., Cunha, H. A., Vidal, L. G., Malm, O. Registros de encalhes de individuos vivos da especie *K. sima* (Owen, 1866) o cachalote-anão, no litoral do Estado do Rio de Janeiro, com notas ecotoxicologicas. *In I Congresso Brasileiro de Biologia Marinha*; 2006 May 15-19; UFF, Niterói, Rio de janeiro, Brazil. p. 101.

14. Zampirolli, E., Alvarenga, F. S., Vicente, A. F. C. Registros de cetáceos e pinípedes para a região da Baixada Santista, São Paulo - Brasil, no período de 1997/2000. *In IX Reunión de Trabajo de Especialistas em Mamíferos Acuáticos de América del Sur*; 2000 Oct 30 – Nov 3; Buenos Aires, Argentina. p. 139.

15. Marcondes, M. C. C., Luna, F. O., Lima, R. P. Rescue and care of a neonate dwarf sperm whale (*Kogia simus*) predated by a cookiecutter shark (*Isistius braziliensis*). In *Proceedings of the Florida Marine Mammal Health Conference*; 2002 Apr 04-07; Gainesville, Florida, USA.

16. Colares, F. A. P., Flach, L., Flach, P. A. Primeiro registro do cachalote-anão, *Kogia sima* (Owen, 1866) no estado do Rio de Janeiro, Brasil. *II Congresso Brasileiro de Mastozoologia*; 2003 Jun 26-29; Belo Horizonte, Minas Gerais, Brazil. p. 54-55.

17. Estima, S. C., Silva, K. G., Monteiro, D. S. Ocorrência de cetáceos no litoral do Rio Grande do Sul, Brasil, entre 1998 e 2001. *In IV Congresso de la Sociedad Latino Americana de Especialistas em Mamíferos Acuaticos*; 2002 Oct 14-19; SOLAMAC, Valdivia, Chile. p. 80-81.

18. Souto, L. R. A., Abrão-Oliveira, J. G., Nunes, J. de A. C. da C., Maia-Nogueira, R., and Sampaio, C. L. S. (2007). Análise das mordidas de tubarões-charuto, *Isistius* spp. (Squaliformes: Dalatiidae) em cetáceos (Mammalia: Cetacea) no litoral da Bahia, Nordeste do Brasil. *Biotemas* 20, 19–25.

19. Souto, L. R. A., Lemos, L. M., Violante, T. H. A. S., and Maia-Nogueira, R. (2009). Record of a neonate dwarf sperm whale, *Kogia sima* (Owen, 1866) stranded on the coast of Bahia, northeastern Brazil. *Lat. Am. J. Aquat. Mamm.* 7, 105–106.

20. Carvalho, C. T. (1966). Notas sobre *Kogia breviceps* (Cetacea, Physeteridae). *Rev. Biol. Trop. 14*, 169-181.

21. Geise, L., Borobia, M. (1988). Sobre a ocorrência de cetáceos no litoral do estado do Rio de Janeiro, entre 1968 e 1984. *Rev. Bras. Zool. 4*, 341-346.

22. Rosas, F. C. W., Pinedo, M. C. (1989). Nota sobre a ocorrência de cachalote pigmeu, *Kogia breviceps*, no litoral do Rio Grande do Sul, Brasil. *Atlântica 11*, 109-113.

23. Santos, C. P., and Lodi, L. (1998). Occurrence of *Anisakis physeteris* Baylis, 1923 and Pseudoterranova sp. (Nematoda) in pygmy sperm whale *Kogia breviceps* (De Blainvillei, 1838) (Physeteridae) in northeastern coast of Brazil. *Mem. Inst. Oswaldo Cruz* 93, 187–188.

24. Muñoz-Hincapié, M. F., Mora Pinto, D. M., Palacios, D. M., Secchi, E. R., Mignucci-Giannoni, A. A. (1998). First osteological record of the dwarf sperm whale in Colombia, with notes on the zoogeography of *Kogia* in South America. *Rev. Acad. Colomb. Cienc. 22*, 433-444.

25. Schmiegelow, J. M. M. (1990) *Estudo sobre cetáceos odontocetos em praias da região entre Iguape (SP) e Baía de Paranaguá (PR) (24º42'S-25º28'S), com especial referência a* Sotalia fluviatilis *(Gervais, 1853) (Delphinidae)* (Master thesis). São Paulo: USP - Universidade de São Paulo.

26. Secchi, E. R., Campolim, M. B., Möller, L. M. Nota sobre o encalhe de dois cachalotes pigmeus *Kogia breviceps* na costa sul do Rio Grande do Sul, Brasil. *In IV Reunión de Trabajo de Especialistas en Mamíferos Acuáticos de América del Sur*; 1994 Oct 24-28; Valdivia, Chile. p. 244-262.

27. Moreira, M. B., Dhremer, C. J., Ferigolo, J. Sobre a ocorrência de *Kogia breviceps* no litoral do Rio Grande do Sul, Brasil, contribuição à sistemática. *In VII Reunión de Trabajo de Especialistas en Mamíferos Acuáticos de América del Sur;* 1996 Oct 22-25; Viña del Mar, Chile. p. 52.

28. Martuscelli, P., Olmos, F., Silva, S. R., Mazzarella, I. P., Pino, F. V., Raduan, E. N. (1996). Cetaceans of São Paulo, Southeastern Brazil. *Mammalia 60*, 125-139.

29. Soto, J. M. R., Ternes-Silva, S. Novos registros de *Kogia breviceps* e *Kogia simus* (Cetacea, Physeteridae, kogiinae) no sul do Brasil e revisão dos registros em águas brasileiras. In *XI Semana Nacional de Oceanografia*; 1998; Rio Grande, Brazil. p. 270-272

30. Carvalho, R. V., Messias, L. T., Silva, K. G. (1998) Ocorrência de cetáceos no litoral do Rio Grande do Sul - (RS) – Brasil 1995/1996/1997. *In VIII Congresso de la Sociedad Latino Americana de Especialistas em Mamíferos Acuaticos*; 1998 Oct 25-29; SOLAMAC, Pernambuco, Brasil. p. 45.

31. Marrano, A., Vicente A. F. C. Registros de encalhes de espécimes do gênero *Kogia* no litoral Central de São Paulo de 1998-2008. In *XII Reuníon de Trabajo de Especialistas en Mamíferos Acuáticos de América de Sur*; 2008 Oct 13-17; Montevideo, Uruguay. p. 167.

32. Medeiros, P. I. A. P. (2006) *Encalhes de cetáceos ocorridos no período de 1984 a 2005 no litoral do Rio Grande do Norte, Brasil (Master thesis)*. Natal, Rio Grande do Norte : UFRN - Universidade Federal do Rio Grande do Norte.

33. Groch, K. R., Marcondes, M. C. C. Impactação gástrica devido à ingestão de plástico por um cachalote pigmeu (*Kogia breviceps*). *In I Reunión Internacional sobre el Estudio de los Mamíferos* Acuáticos; 2006 Nov 05-09; SOMMEMA/SOLAMAC, Mérida, Mexico. p. 12.

34. Brito, A. P. D., Bauer, L. M., Veloso, R. S. Registro, avaliação hematológica e urinálise de dois espécimes de cachalote-pigmeu, *Kogia breviceps* (Blainville, 1838) encalhados no litoral da Bahia, nordeste do Brasil. In V*I Encontro Nacional Sobre Conservação e Pesquisa de Mamíferos Aqüáticos*; 2009 Nov 09-14; ENCOPEMAQ, Salvador, Bahia, Brazil. p. 44.

35. Alves, A. K. M., Sartori, C. M., Schulze, B., Holz, A. C., Santos, N. Z., Paitach, E. L., Cremer, M. J. (2013) Ocorrência do gênero *Kogia* (Cetartiodactyla, Kogiidae) em Santa Catarina. *In IV Congresso Brasileiro de Biologia Marinha*; 2013 May 19-23; Santa Catarina, Brazil.
