# Supplementary material for: Stranding Events of Kogia Whales along the Brazilian Coast
Source: PLoS One. 2016 Jan 5;11(1):e0146108. doi: 10.1371/journal.pone.0146108 (PMC4701718; doi:10.1371/journal.pone.0146108)
Supplement: S2 Table — Reported are parameter estimates (± standard error) for intercept “α” and slope “β” of each model. Corresponding p-values are p < 0.05 for *, p < 0.01 for **, and p < 0.001 for ***. (DOCX) [file pone.0146108.s002.docx]

**S2 Table. Summary statistics of the Generalized Linear Model method for the single effect of each environmental variable on *Kogia* strandings.** Reported are parameter estimates (± standard error) for intercept “α” and slope “β” of each model. Corresponding p-values are p < 0.05 for *, p < 0.01 for **, and p < 0.001 for ***.

| **Predictor** | **Parameter** | **TN** | **KB** | **KS** |
| --- | --- | --- | --- | --- |
| Chl-a | **α**Chl-a | 1.41100 ± 0.52250** | -0.23710 ± 0.46030* | 1.44180 ± 0.57380 |
|  | **β**Chl-a | 4.02420 ± 2.50920 | 7.4584 ± 1.8153*** | 1.17240 ± 2.80610 |
| SST | **α**SST | 4.02509 ± 2.66652 | 7.09150 ± 2.30470** | 0.34233 ± 2.81471 |
|  | **β**SST | -0.07168 ± 0.10430 | -0.23040 ± 0.09220* | 0.05146 ± 0.10951 |
| Wspe | **α**Wspe | -4.01370 ± 2.09400* | -6.01880 ± 2.10840** | -3.05930 ± 2.46010 |
|  | **β**Wspe | 0.87500 ± 0.29470** | 1.02710 ± 0.28840*** | 0.66770 ± 0.34670**˙** |
| Wdir | **α**Wdir | 2.02829 ± 2.02156 | -0.52564 ± 2.34171 | 2.57115 ± 2.02743 |
|  | **β**Wdir | 0.00099 ± 0.01112 | 0.01022 ± 0.01279 | -0.00506 ± 0.01120 |
| Depth | **α**depth | 2.91934 ± 0.58468* | 2.67842 ± 0.58898*** | 1.97573 ± 0.63120** |
|  | **β**depth | 0.00032 ± 0.00023 | 0.00063 ± 0.00026* | 0.00013 ± 0.00025 |
