# Supplementary material for: Stranding Events of Kogia Whales along the Brazilian Coast
Source: PLoS One. 2016 Jan 5;11(1):e0146108. doi: 10.1371/journal.pone.0146108 (PMC4701718; doi:10.1371/journal.pone.0146108)
Supplement: S3 Table — The selection is based on the Akaike’s Information Criterion (AIC) and Akaike’s Weights (WAIC). The best models are the one that have the lowest AIC and highest WAIC, here highlighted in bold and with superscript (1). The second and third subsequent most important models according to AIC are indicated with superscripts (2) and (3), respectively. (DOCX) [file pone.0146108.s003.docx]

**S3 Table. Model selection from a series of additive models.** The selection is based on the Akaike’s Information Criterion (AIC) and Akaike’s Weights (WAIC). The best models are the one that have the lowest AIC and highest WAIC, here highlighted in bold and with superscript (1). The second and third subsequent most important models according to AIC are indicated with superscripts (2) and (3), respectively.

| **Models** | **TN** | | **KB** | | **KS** | |
| --- | --- | --- | --- | --- | --- | --- |
|  | AICs | W_AIC_ | AICs | W_AIC_ | AICs | W_AIC_ |
| M1= Chl-a | 73.424 | 2.162 | 48.225 | 3.729 | 64.041 | 2.631 |
| M2= SST | 75.176 | 0.900 | 53.546 | 0.261 | 63.977 | 2.717 |
| M3= W_spe_ | 69.991^(2)^ | 12,03^(2)^ | 49.138 | 2.362 | 61.452^(4)^ | 9.602^(4)^ |
| M4= W_dir_ | 75.669 | 0.704 | 63.761 | 0.002 | 63.987 | 2.703 |
| M5= Depth | 74.061 | 1.573 | 55.739 | 0.087 | 63.916 | 2.801 |
| M6= Chl-a+ SST | 74.733 | 1.124 | 50.214 | 1.379 | 64.252 | 2.368 |
| M7= Chl-a+ W_spe_ | 70.988^(4)^ | 7.31^(4)^ | **45.666^(1)^** | **13.399^(1)^** | 63.408 | 3.612 |
| M8= Chl-a+ W_dir_ | 74.250 | 1.43 | 49.541 | 1.930 | 65.219 | 1.460 |
| M9= Chl-a+ Depth | 75.380 | 0.813 | 50.111 | 1.452 | 65.914 | 1.031 |
| M10= SST + W_spe_ | 71.582 | 5.43 | 50.052 | 1.495 | 61.388^(3)^ | 9.913^(3)^ |
| M11= SST + W_dir_ | 76.882 | 0.384 | 55.359 | 0.105 | 65.941 | 1.017 |
| M12= SST + Depth | 76.051 | 0.581 | 54.224 | 0.186 | 64.654 | 1.936 |
| M13= W_spe_ + W_dir_ | 71.707 | 5.102 | 50.608 | 1.133 | 63.097 | 4.217 |
| M14= Wspe + Depth | 70.957^(3)^ | 7.423^(3)^ | 45.704^(2)^ | 13.149^(2)^ | 63.450 | 3.535 |
| M15= Wdir + Depth | 75.293 | 0.849 | 57.672 | 0.033 | 65.000 | 1.629 |
| M16= Chl-a+ SST + W_spe_ | **69.709^(1)^** | **13.852^(1)^** | 46.460^(4)^ | 9.011^(4)^ | 61.384^(2)^ | 9.935^(2)^ |
| M17= Chl-a+ SST + W_dir_ | 76.096 | 0.568 | 51.529 | 0.715 | 66.091 | 0.944 |
| M18= Chl-a+ SST + Depth | 76.653 | 0.43 | 52.104 | 0.536 | 65.983 | 0.996 |
| M19= Chl-a+ W_spe_ + W_dir_ | 72.366 | 3.669 | 47.570 | 5.173 | 65.051 | 1.588 |
| M20= Chl-a+ Wspe + Depth | 72.877 | 2.842 | 46.412^(3)^ | 9.228^(3)^ | 65.292 | 1.407 |
| M21= Chl-a+ Wdir + Depth | 76.006 | 0.595 | 51.137 | 0.869 | 66.929 | 0.621 |
| M22= SST + W_spe_ + W_dir_ | 73.507 | 2.075 | 52.036 | 0.555 | 62.985 | 4.462 |
| M23= SST + Wspe + Depth | 71.117 | 6.852 | 47.551 | 5.222 | **61.336^(1)^** | **10.175^(1)^** |
| M24= SST + Wdir + Depth | 77.168 | 0.333 | 55.652 | 0.091 | 66.412 | 0.804 |
| M25= Wspe + Wdir + Depth | 72.412 | 3.586 | 47.296 | 5.934 | 64.856 | 1.751 |
| M26= Chl-a+ SST + W_spe_ + W_dir_ | 71.991 | 4.426 | 48.450 | 3.331 | 63.143 | 4.122 |
| M27= Chl-a+ SST + Wspe + Depth | 71.291 | 6.281 | 46.667 | 8.126 | 62.720 | 5.094 |
| M28= Chl-a+ SST + Wdir + Depth | 77.848 | 0.237 | 53.130 | 0.321 | 67.722 | 0.418 |
| M29= Chl-a+ Wspe + Wdir + Depth | 74.092 | 1.548 | 47.762 | 4.699 | 66.832 | 0.652 |
| M30= SST + Wspe + Wdir + Depth | 73.098 | 2.545 | 49.269 | 2.212 | 63.254 | 3.899 |
| M31= Chl-a+ SST+ Wspe + Wdir + Depth | 73.260 | 2.347 | 48.483 | 3.276 | 64.631 | 1.959 |
